# Mechanical behavior and particle crushing of irregular granular material under high pressure using discrete element method

**DOI:** 10.1038/s41598-023-35022-w

**Published:** 2023-05-15

**Authors:** Qinglin Chen, Zugui Li, Zeyu Dai, Xiaojun Wang, Chao Zhang, Xuepeng Zhang

**Affiliations:** 1 School of Resources and Environmental Engineering, Jiangxi University of Science and Technology, Ganzhou, 341000 Jiangxi China; 2 State Key Laboratory of Geomechanics and Geotechnical Engineering, Institute of Rock and Soil Mechanics, Chinese Academy of Sciences, Wuhan, 430071 Hubei China

**Keywords:** Civil engineering, Mechanical engineering

## Abstract

This study investigated the influence of stress levels on the mechanical behavior and particle crushing of irregular granular materials. Granular materials with irregular sides were modelled using the discrete element method. A new method of using a shear fracture zone to characterize the deformation of irregular granular materials under high pressure was proposed. The crushing energy is analysed based on the first law of thermodynamics. The shear strength of irregular granular materials shows significantly nonlinear behavior due to particle crushing. The deformation behavior can be characterized with the help of particle rotation under low confining pressure, and can be characterized with the help of particle breakage under high confining pressure. Granular materials easily break into many single fine particles under high confining pressure. The breakage degree can be represented by the value of crushing energy. Irregular granular materials have a large breakage degree under high confining pressures. It weakens the stability of engineered structures constructed from granular materials.

## Introduction

With rapid developments in industrialization, numerous high earth-rock dams, waste rock piles and tailing dams have been built worldwide. The particle skeletons of these engineering structures are granular materials. In China, earth-rock dams, waste rock piles and tailing dams have reached heights of 300 m, 350 m, and 200 m, respectively. The stress in such high dams or piles increases with height. One of the most significant concerns for granular materials is particle breakage under high pressure. The degree of particle breakage increases with an increasing stress level^1,2^. The macroscopic behavior of granular materials is largely affected by the degree of particle breakage, such as the stress–strain relationship, shear strength, volumetric strain, and yielding^3,4^. The reason is that the change in the particle size distribution may cause an extremely drastic change in the internal structure of granular materials. Therefore, particle breakage plays an important role in the stability of engineering structures constructed with granular materials, especially under high pressure. For example, on 2007, the Canoas Novos rock-fill dam with the height of 202 m in Brazil suffered overall destruction at the bottom of the dam due to particle breakage^5^. The Nam Ngum 2 rockfill dam in Thailand suffered a 24.6 cm (or 0.15% of dam height) dam crest settlement due to particle breakage during only 3 years of reservoir operation^6^.

Particle breakage is the damage or breaking of particles, and some fine particles occurred during loading^7,8^. Many factors affect the particle crushing of granular materials, such as relative density, particle size, particle shape, etc.^9,10^. The effect of particle crushing on internal friction angle of rockfill materials was studied by Gamboa^11^, who found that the internal friction angle decreases with the increase in the amount of particle crushing. To study the impact of particle crushing on the critical state line, the triaxial shear tests were conducted by Bandini and Coop^12^. Their results show that the critical state line moves vertically downward due to the particle breakage. The particle breakage reduces the porosity of granular materials, which causes a shift in the critical state line that includes both an offset and a rotation^13^. The particle crushing of calcareous sand was investigated by Wu et al.^14^, and found that excessive deformation and foundation failure occur as a result of particle breakage.

The Discrete Element Method (DEM) was first described by Cundall and Strack^15^. The DEM has some advantages in the study of the micromechanical mechanisms of the granular materials. The laboratory test results can be reproduced inexpensively. Therefore, the micromechanics caused by particle crushing have received more attention from several researchers due to the non-reproducibility of the particle crushing tests^16–18^. In DEM, two possible approaches were used to describe particle crushing. In one approach, the particle agglomerates were bonded by parallel bond model. The process of particle crushing relies on the bond strength of particles in this approach^19,20^. For the second approach, the grains are replaced by finer fragments when they exceed the failure criterion. A proper breakage criterion is defined based on the particle size and the contacts around them in the second approach^21–23^. The two methods have been extensively used to study the particle breakage, especially for sharp-edge particles. The crushable granular materials were modelled using DEM methods^24,25^. Some coupling methods were also used to study the particle crushing of granular materials, such as DEM-FEM, DEM-XFEM and DEM-SBFEM^26–28^. The numerical method has brought the investigation of particle breakage into a grain scale and provided several new insights. Liu et al.^29^ investigated the relationships between particle breakage and intermediate principal stress. Bono and McDowell^30^ studied the influence of particle crushing on the critical states of crushable sand using DEM methods. The evolution of particle shape of granular materials during the crushing was simulated by Bisht and Das^31^. The DEM method was used to simulate the wetting deformation of rockfill materials under different stress paths by Shao et al.^32^. In fact, for the granular materials, the particle shape of the aggregates is irregular and randomly distributed. To precisely describe the mechanical behavior of granular materials, a suitable model which is able to simulate arbitrarily shaped particles should be pay more attention.

This paper aims to show the mechanical properties of crushable granular materials from a mesoscopic perspective. Based on the principles of irregularly shaped polygon modelling, particle clusters with irregular sides were modelled using a DEM with some particles. A biaxial numerical model test was carried out under a high confining pressure. The impact of the stress level on the mechanical behavior and particle crushing of granular materials was studied. A new method of using the shear fracture zone to characterize the deformation of granular materials is proposed. The evolution of the microcracks caused by particle crushing was investigated. The crushing energy in the shear process is analysed according to the first law of thermodynamics.

## Modelled irregularly shaped polygons

Two types of nonspherical particle units are used in the numerical simulation: (1) particle clumps and (2) particle clusters^33^. A particle clump can simulate irregular granular materials. Clumps are made of spheres that are rigidly connected, giving the final shape to the particle. The breakage does not occur. A particle cluster is composed of some spheres. The spheres are joined together by parallel bonds. Such parallel bonds are characterized by the tensile strength and cohesion, allowing for breakage during the simulation. Therefore, the particle cluster can not only simulate irregular granular materials but also simulate particle crushing. The realistic mechanical characteristics of granular materials can be simulated by particle clusters, especially under high confining pressures.

**Figure 1:**
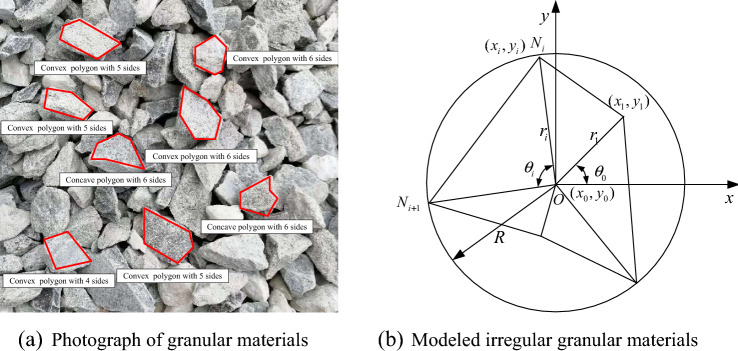
Irregular granular materials.

Figure 1a presents a photograph of the granular materials. Granular materials are random irregular polyhedrons. The boundary of the granular materials can be simplified to polygons in 2-D numerical simulations^34,35^. Most granular materials are convex polygons, and a few granular materials are concave polygons. To more accurately characterize the actual behavior of granular materials, the concave and convex polygons are modelled. The Monte Carlo method is used to model particle clusters because the particle shape and particle size are random^36^. Specifically, an irregular polygon is modelled in a circle with certain particle size. Figure 1b presents a schematic diagram of the modelled irregular granular materials. The detailed model process can be given as follows^37^:

1. Determination of the side number $n$

The edge number $n$ is calculated by Eq. (1) as follows:

1 $$n = \delta \cdot (b - a) + a$$

where $n$ is the side number of the polygon, which is automatically rounded by the computer system; $\delta$ is a random number, which is uniformly distributed in the range of 0–1; and $a$ and $b$ are the minimum and maximum edge numbers in the granular assembly, respectively.

(B) Determination of the central angle $\theta_{i}$

The central angle $\theta_{i}$ corresponding to the *i*-th side is calculated by Eq. (2) as follows:

2 $$\theta_{i} = 2\pi \cdot \eta_{i} /\sum\limits_{{1}}^{n} {\eta_{i} }$$

where $\eta_{i}$ is a random number, which can be calculated by Eq. (3) as follows:

3 $$\eta_{i} = \delta (1 - S) + S$$

where $S$ is the smoothness of the polygon, which is in the range of 0 to 1. When $S$ is near one, the values between the central angles $\theta_{i}$ are approximately equal, indicating that the generated polygon is round. When $S$ is near zero, there are large differences between the central angles $\theta_{i}$, indicating that the generated polygon is very irregular.

(C) Determination of span $r_{i}$

The span $r_{i}$ can be calculated by Eq. (4) as follows:

4 $$r_{i} = AR + (1 - A)\delta R$$

where $R$ is the circumscribed circle radius; and $A$ is the shape factor, which is in the range of 0 to 1. The vertices of the polygon are near the boundary of the circumcircle when $A$ is near one and $A$ is near zero, indicating that the vertices are created inside the circumcircle. Therefore, the concave and convex polygons are modelled by Eq. (4).

(D) Determination of the vertices coordinate

The vertex coordinates of the polygon can be calculated by Eq. (5) as follows:

5 $$\left. {\begin{array}{*{20}c} {x_{i} = x_{0} + r_{i} \cos \theta^{\prime}_{i} } \\ {y_{i} = y_{0} + r_{i} \sin \theta^{\prime}_{i} } \\ \end{array} } \right\}$$

where $x_{0}$, $y_{0}$ is the coordinate of the circle centre; and $\theta^{\prime}_{i} = \sum\nolimits_{{1}}^{i} {\theta_{i} } + \theta_{0} - \theta_{i}$, $\theta_{0} \in (0,2\pi )$, is the starting angle.

In this paper, the values of *a* and $b$ were set to 4 and 6, respectively, which indicates that the minimum side number of the modelled irregular polygon is 4, and the maximum number of sides is 6. The smoothness $S$ and shape factor $A$ are the custom parameter. In general, the shape coefficient is in range of 0.4–0.8 for the rockfill materials^38^, and is in range of 0.57–0.76 for the sand^39^. The smoothness $S$ and the shape factor $A$ of the granular materials were both set to 0.7 in this paper.

## Modelled methodology

### DEM method

The commercial PFC^2D^ software was used to perform the DEM numerical simulation. The computing efficiency in 2-D the is higher than that in 3-D. The soft contact approach is used in the PFC^2D^ software. The particles are bonded together. The particles are joined together by parallel bonds. The parallel-bond component acts in parallel with the linear component and establishes an elastic interaction between the particles. The existence of a parallel bond does not preclude the possibility of slip. Particles interact at pair-wise contacts by means of an internal force and moment by parallel bond model. The parallel bond Normal stiffness is 2e^8^ N/m, and the bond normal-to-shear stiffness ratio is 1. In general, the granular material is a material without cohesion. They are irregular, with distinct angularity. Therefore, a linear model^40^ and rolling resistance linear model^41,42^ were assigned to the granular materials.

### Specimen preparation

**Figure 2:**
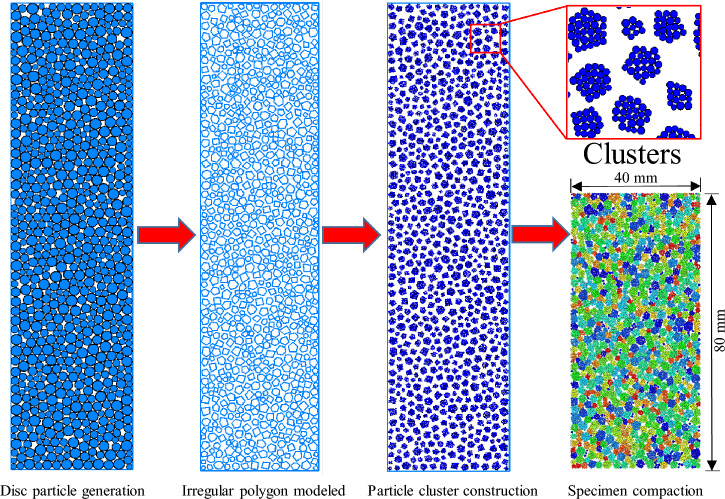
Flow diagram of specimen preparation.

Irregular polygons may intersect when the polygon is created using the random centre coordinates method^43^. Therefore, the contact detection algorithm needs to identify whether the generated polygons intersect. However, this method is expensive because the discrimination process is cumbersome^44,45^. To overcome intersection of the generated polygon, irregular polygons were created with the aid of nonoverlapping disc particles in this paper^46,47^. Figure 2 presents the flow diagram of specimen preparation. Additional details on the specimen preparation process can be given as follows:

1. *Disc particle generation* Disc particles with a minimum radius of 1 mm and maximum radius of 2 mm are generated in space with a width of 40 mm and height of 200 mm. The initial porosity is 0.1, and 785 particles are generated.
2. *Irregular polygon modelling* Irregular polygons were modelled in each disc particle according to the random polygon construction principle. The disc particles were deleted after the polygon was created. The polygons generated do not intersect because the disc particles do not overlap. The boundary of the irregular polygon is also the boundary of the particle cluster.
3. *Particle cluster construction* The particles are generated inside the irregular polygon. The radius of the particles ranges from 0.25 to 0.3 mm with a uniform distribution.
4. *Specimen compaction and pore filling* The specimen is compacted after the particle clusters are generated because the pores between the particle clusters are large. After the model is compacted, the particle clusters whose height is greater than 80 cm are deleted. For a few large pores which are exist between the particle clusters, they are filled with disc particles. The radius of the filled particles ranges from 0.3 to 0.5 mm with a uniform distribution.

The height of the samples was 80 mm and width was 40 mm. The total number of particle clusters is 763; the minimum number of particles contained in clusters is 5; and the maximum number of particles is 30. The total number of particles is 10,985. The linear model and rolling resistance linear model were assigned to the samples. Ten micro-mechanical parameters are required to carry out a DEM simulation. The micromechanical parameters in the present paper are shown in Table 1. The micro-mechanical parameters were calibrated through the experimental results of the high pressure triaxial shear tests conducted on the medium-dense tailings sand with the dry density of 1.6 g/cm^3^. The four main components are in the apparatus the control system, the triaxial pressure chamber, the motor and the confining pressure system, respectively. The wall thickness of the triaxial pressure chamber is 12 mm. It is made of stainless steel. The maximum confining pressure of the apparatus is 5 MPa. The maximum axial load is 60 kN.

**Table. 1:** Micro-mechanical parameters in the specimens.

	Linear normal contact stiffness (N/m)	Normal-to-shear stiffness ratio	Friction coefficient	Parallel bond normal stiffness (N/m)	Bond normal-to-shear stiffness ratio	Parallel bond strength (N/m)	Parallel bond cohesion (N/m)	Parallel bond friction angle (°)	Rolling resistance coefficient	Parallel-bond moment (N m)
Particles in cluster	1e8	1	0.3	2e8	1	3.5e6	5e6	35		
Clusters	1e8	1	0.4						0.8	20

**Figure 3:**
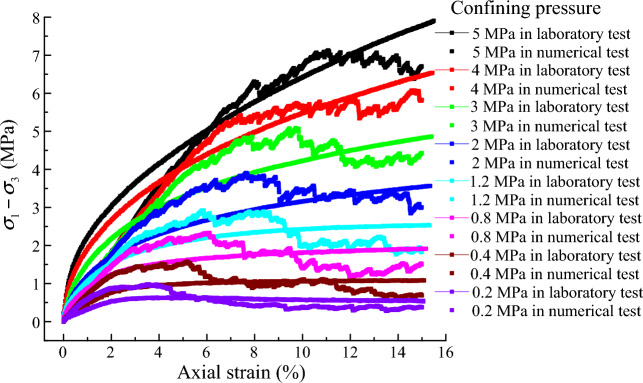
Stress–strain relationship curves of the samples.

Figure 3 presents the comparison of stress–strain curves between the numerical and experimental tests. The simulated curves are wave-varying and the tested curves are monotonous. The results of DEM simulations agree generally with the experimental results. Therefore, it is relatively reliable that the numerical model was established and the micro-mechanical parameters were calibrated in this paper.

### Test program

**Figure 4:**
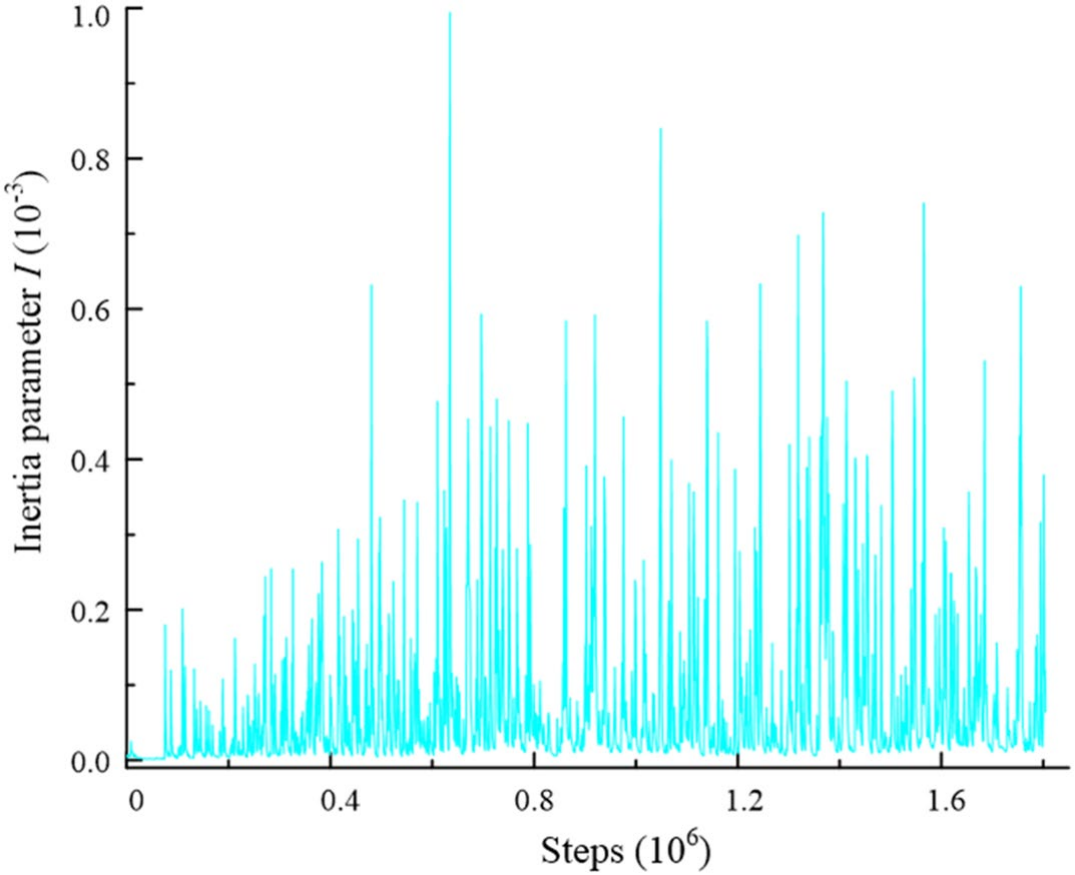
Inertia parameter* I.*

A series of numerical drained biaxial tests were carried out in this study. The stiffness of the top plates, bottom plates and side walls were both set to 1e^8^ N/m, and the friction coefficients of all walls were set to 0. The shear speed of 0.01 m/s was applied to the top and bottom plates which moved towards each other. The strain-controlled was performed when the samples were sheared. The constant confining pressure were maintained by the side walls which were applied to servo mechanism. The kinetic energy generated during shearing must be negligible. Otherwise, the calculation of the model will be invalid. Therefore, the shear rate should be as slow as possible. To ensure quasi-static shearing, an inertia parameter *I* was proposed by Da Cruz et al.^48^ and Gong and Liu^49^, as follows:

6 $$I = \dot{\varepsilon }\sqrt{\frac{m}{p}}$$

where $\dot{\varepsilon }$ is the axial strain rate; $p$ is the mean pressure; and $m$ is the particle mass. The quasi-static shearing was ensured when $I\ll 1$. Figure 4 presents the inertia parameter *I* in this simulation. The inertia parameter *I* is less than 10^–3^ during shearing. The quasi-static shearing in this simulation were satisfied. The computational efficiency and accuracy were reasonable in the DEM simulation. The results in this paper can be considered reliable.

**Figure 5:**
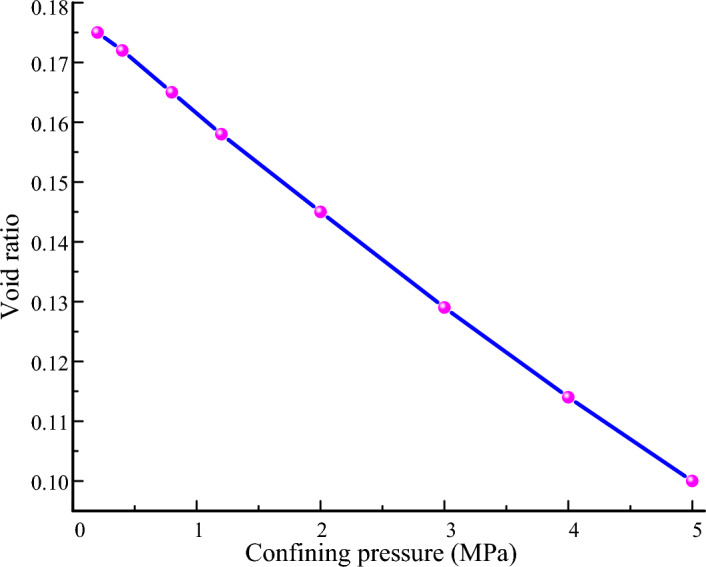
Void ratio of sample at end of applying confining pressure.

With the construction of large projects, such as high dams, high storage areas, and high buildings, the mechanical behavior of granular materials under the high stress has received particular attention. Therefore, two levels of confining pressure are used in this paper. The low confining pressures of the test are 0.2 MPa, 0.4 MPa, and 0.8 MPa. The high confining pressures of the test are 1.2 MPa, 2 MPa, 3 MPa, 4 MPa, and 5 MPa. Figure 5 presents the void ratio of sample at end of applying confining pressure. The void ratio decreases linearly with an increase in the confining pressure. The shear test is terminated when the axial strain reaches 15%.

## Mechanical properties of granular material

### Stress–strain behavior

**Figure 6:**
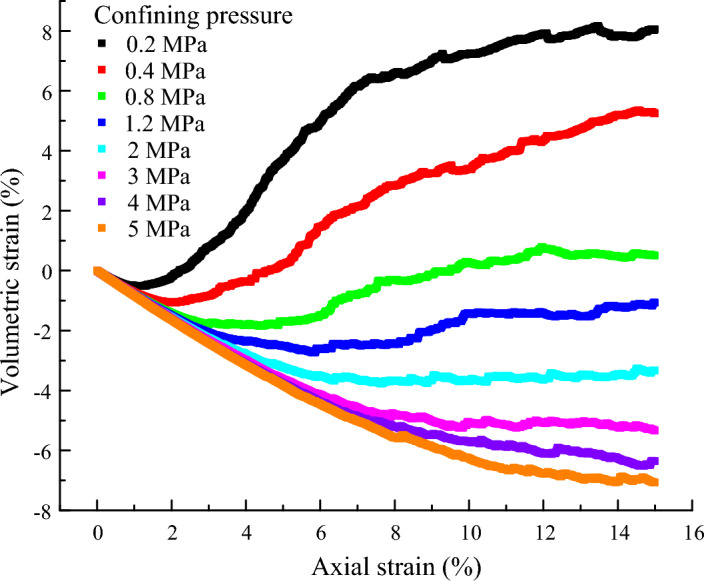
Volumetric strain of the samples.

The stress in the small axial strain increases monotonously with increasing axial strain, and a stiff response is exhibited at the initial stage, as shown in Fig. 3. When the axial strain is large, the stress–strain curves show more wave-varying. All shear strengths and axial strain at the peak increase with an increase in the confining pressure. Figure 6 presents the volumetric strain of the samples. For the samples under the confining pressure less than 1.2 MPa, the volumetric strain first decreases until it reaches a minimum and then increases with increasing axial strain. A volumetric dilation occurs in these samples. For the samples under confining pressures greater than 1.2 MPa, the volumetric strain decreases with increasing axial strain. A volumetric contraction occurs in these samples.

**Figure 7:**
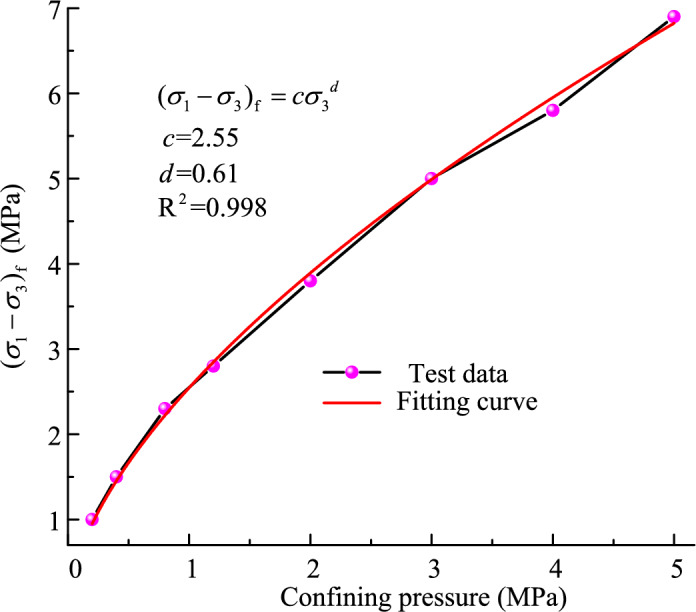
Relationship between shear strength and confining pressure.

Figure 7 presents the relationship between the shear strength and the confining pressure. The values of the shear strength are measured from Fig. 3. The shear strength shows a significantly nonlinear behavior. This is attributed to both particle crushing and stress level^50^. A nonlinear strength mathematical model proposed by Yin et al.^51^ can be given as follows:

7 $$(\sigma_{1} - \sigma_{3} )_{{\text{f}}} = c\sigma_{{3}}^{d}$$

where *c* and *d* are the tested constants. Constant *c* denotes the linear growth rate of shear strength. Constant *d* denotes the nonlinear growth rate. The shear strength data is fitted by Eq. (7). The fitting curve is in good agreement with the shear strength when comparing the fitted curves with the shear strength data. Therefore, Eq. (7) can be characterized the nonlinear strength relationship of the granular material.

### Deformation behavior

**Figure 8:**
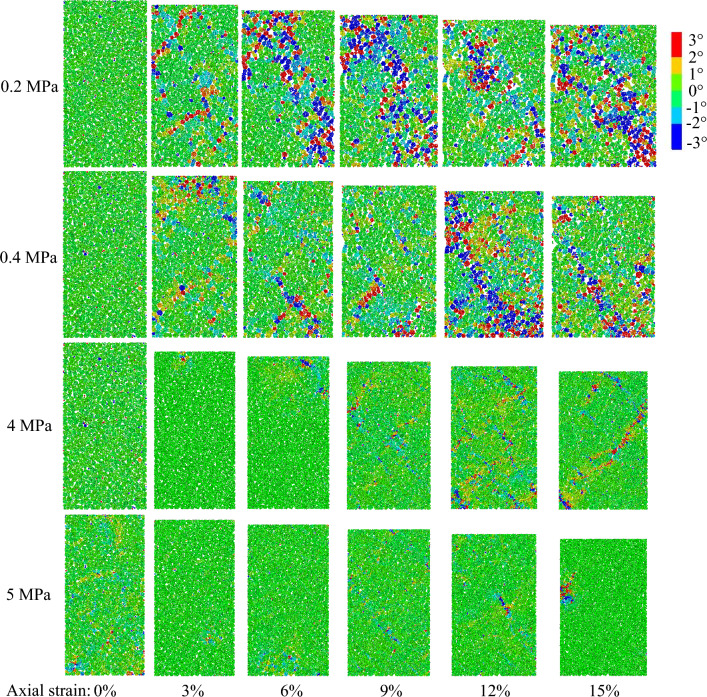
Particle rotation cloud in samples.

**Figure 9:**
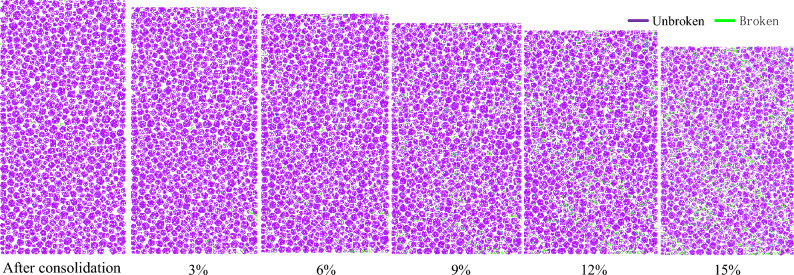
Contact fracture of the sample under confining pressure of 4 MPa.

When granular materials shear under low confining pressures, localized shear banding is prone to form in the stress concentration zone due to particle rotation^52^. Therefore, particle rotation can be characterized by the deformation of granular materials under the low confining pressure. To study deformation caused by confining pressure, samples under the confining pressure of 0.2 MPa, 0.4 MPa, 4 MPa and 5 MPa were selected. Figure 8 presents the particle rotation cloud in the samples. For samples under the low confining pressure of 0.2 MPa and 0.4 MPa, the particle rotation gradually becomes obvious with an increasing axial strain. Macroscopic shear banding at a dip angle of 45° occurs in these samples. The particle clusters in macroscopic shear banding are loose, and the pores are large. The shear dilatation occurred in the samples. The width of the shear banding increases with increasing axial strain. The rotation angles of particles in the same cluster are equal due to the fact that the particles are bonded together with parallel bonds. For samples under the high confining pressure of 4 MPa and 5 MPa, the distribution of particle clusters in the sample is dense, and the porosity between the particle clusters is small. The mechanical behavior of the samples is shear contraction. Shear banding does not occur in these samples. Therefore, the deformation behavior is not characterized with the aid of particle rotation. This result is attributed to the fact that the degree of particle crushing is larger than that of particle rotation under high confining pressures. To characterize the deformation behavior of the sample under high confining pressures, Fig. 9 presents the contact fractures of the sample under a confining pressure of 4 MPa. The fracture zone becomes gradually obvious with increasing axial strain. The fracture zone is observed to be in the form of a kite shape. Therefore, particle rotation is capable of characterizing the deformation behavior under low confining pressures. The deformation behavior can be characterized with the aid of contact fractures under the high confining pressure.

### Evolution of contact force chain

**Figure 10:**
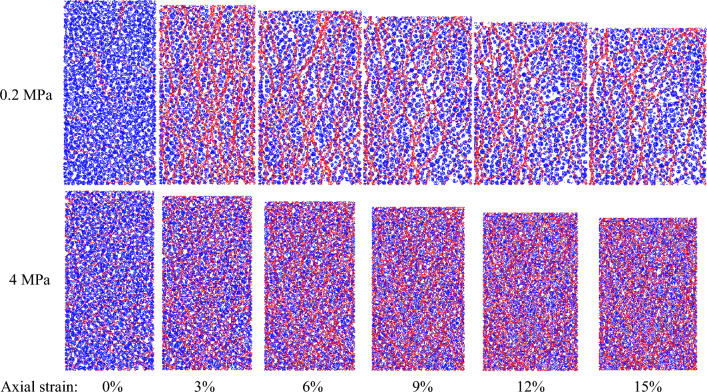
Evolution of the distribution of force chains between particles.

To study the evolution of the force chain caused by confining pressures, samples under the confining pressure of 0.2 MPa and 4 MPa were selected. Figure 10 presents the evolution of the distribution of force chains between particles. Red lines represent a strong contact force, the weak contact force is represented by blue lines. The line width represents the magnitude of contact force. The contact force increases with increasing line width. For the sample under a confining pressure of 0.2 MPa, the threshold, which is the critical transition from the weak contact force to the strong contact force, is 360 N. The number of strong forces first increases and then decreases with increasing axial strain. The distribution of the strong contact force is densest when the axial strain is 3%. The shape of the strong force chain is similar to the dense interweaving grid. For the sample under a confining pressure of 4 MPa, the threshold that is the critical transition from the weak contact force to the strong contact force is 3200 N. The number of strong contact forces increases with increasing axial strain due to the fact that the strain hardening occurs in the samples. The emergence of many strong contact forces to withstand continuously increasing external load. The strong force chains are widely distributed in the sample. When comparing the distribution of the force chains, strong force chains in the specimens under low pressure are sparser than those under high pressure. This indicates that a stronger contact force is formed when the sample is subjected to high external forces. The evolution of the force chains can be quantitatively described by a second order Fourier expansion

8 $$f_{n} (\theta )/f_{0} = 1 + a_{n} \cos 2(\theta - \theta_{n} )$$

where $f_{n} {(}\theta {)}$ are the distribution functions. $\theta_{n}$ are the principal orientation angles. $f_{0}$ is the average contact force. $a_{n}$ is the anisotropy coefficient.

**Figure 11:**
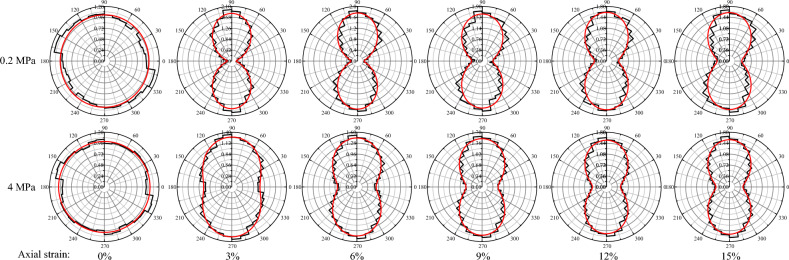
Distributions of normal contact force in polar coordinates.

The statistical distribution of the contact force chain under the confining pressure of 0.2 MPa and the confining pressure of 4 MPa were drawn by calculating the internal contact force of particles under different axial strains. Figure 11 presents the distributions of contact force in polar coordinates. The evolution of contact force is represented by the same normalized scale. The solid red line in Fig. 11 represents the fitting curves corresponding to the Eq. (8). There is a good agreement between Eq. (8) and the data.

At the initial stage (i.e.,$\varepsilon = 0\%$), the distribution shapes of the force chain are approximately circular. It means that the external force applied to the samples are isotropic at the consolidation stage. The distribution of the contact force chains was changed significantly with increasing axial strain. The distribution of the contact force chain converts from a circle to a peanut shape. The principal orientations of the contact forces distribution are always along the axial loading direction (i.e., $\theta_{n} = 90^{ \circ }$).

Comparing the distribution shapes of the contact force, when the axial strain is 3%, the peanut shape drawn by the contact force chain under low pressure is the most oblate, and then with the increase of the axial strain, the peanut shape of contact force distribution is slightly expanded in the lateral direction. The shape of the contact chain under high pressure gradually converts from circular to peanut shape with the increase of axial strain. The peanut shape becomes more oblate. A more interesting finding is that the contact force distribution under low pressure is more oblate than that under high pressure when the axial strain is the same. It indicates that more contact force chains are towards the loading direction under low pressure, especially the strong chains in the sample. Therefore, the most of sample skeleton are used to resist deformation by external forces under low pressure.

**Figure 12:**
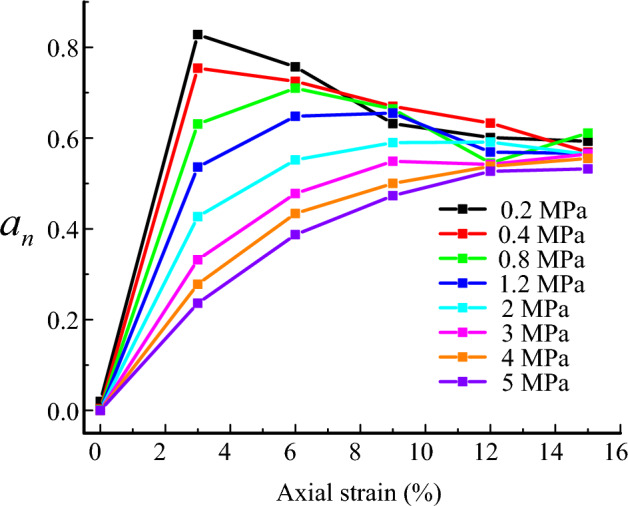
Variation of the anisotropy coefficients $a_{n}$ under the different confining pressure.

Figure 12 presents the variation of the anisotropy coefficients $a_{n}$ under the different confining pressure. When the confining pressure is larger than 2 MPa, the anisotropy coefficients $a_{n}$ increase with axial strain. When the confining pressure is lower than 1.2 MPa, the anisotropy coefficients $a_{n}$ increase, and then decrease with axial strain. The variation trend is similar to that of the stress–strain curve. It seems to indicate that the shear strength of the samples is dominated by the anisotropic coefficient $a_{n}$. In contrast, Gong^53^ reported that $a_{n}$ is the mechanical anisotropic coefficient which affect the shear strength at the peak and critical states. In addition, $a_{n}$ is related to the intensity of the non-homogenous normal contact force proposed by Yang^54^.

## Particle crushing of granular material

### Evolution of contact fracture

The contact fracture of internal adhesion leads to the crushing of particle clusters. Therefore, the crushing characteristics and crushing position of the particle clusters can be judged by identifying the contact state of the cluster particles. To analyse the evolution of contact fracture during the shear test, the sample under the confining pressure of 4 MPa was selected, as shown in Fig. 9. The green line represents that the contact is broken, and the pink line represents that the contact is not broken. A small amount of contact fracture appeared in the sample after consolidation. This is attributed to the extrusion of the edges and corners of the particle clusters, resulting in particle cluster crushing. The fragmentation of particle clusters increases with increasing axial strain during the shearing test. When the axial strain is large, the contact fracture is observed to be in the form of an ‘X’.

**Figure 13:**
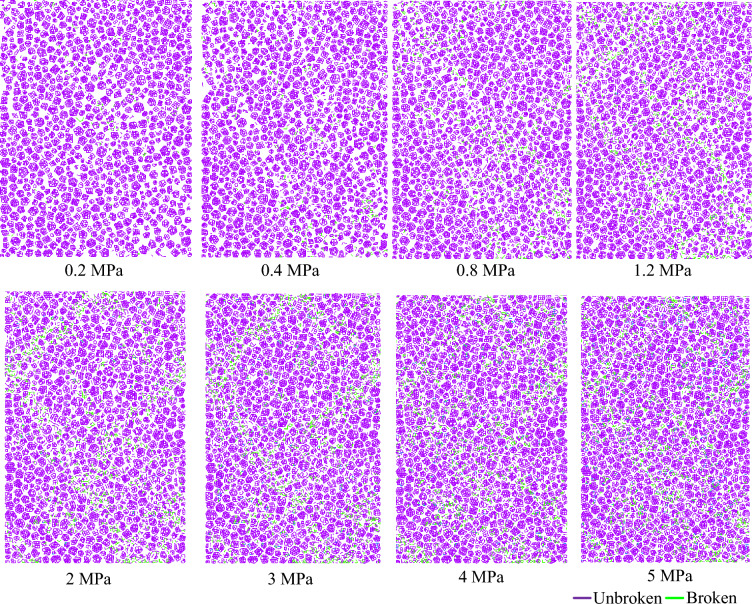
Contact fracture map at the axial strain of 15% under different confining pressure.

To analyse the evolutions of contact fracture caused by confining pressure, a sample deformation at the test end is selected. Figure 13 presents the contact fracture map at the axial strain of 15%. The green line represents that the contact is broken, and the pink line represents that the contact is not broken. The number of contact fractures increases with an increase in the confining pressure. When the confining pressure is less than 0.8 MPa, only a few contact fractures are distributed in the shear banding. There is an obvious broken zone in the sample when the confining pressure is greater than 0.8 MP. The broken zone becomes more obvious with an increase in the confining pressure.

According to deformation results in “Deformation behavior” section, for the samples under the low confining pressure, the movement of the particle clusters is particle rolling in the stress concentration zone. This action results in the movement between particle clusters being unsynchronized. Macroscopic shear banding was generated in the stress concentration zone when the difference in particle rolling between particle clusters was large. For the samples under the low confining pressure, the movement of the particle clusters is dominated by contact fractures in the stress concentration zone. Macroscopic broken banding which is the concentrated zone of fractures is occurred in the stress concentration zone due to perforation of contact fractures. Therefore, the deformation of the samples is transformed from macroscopic shear banding to macroscopic broken banding with an increase in the confining pressure.

### Change in particle size distribution

Particle crushing occurred in the particle clusters, which led to changes in the particle size distribution. Therefore, the change of the particle size distribution before and after the test are able to characterize the degree of particle crushing. In numerical tests, particle size distributions were counted by the self-compiled function. Assuming that the *m*-th particle cluster contains *n* (*n* ≥ 1) particles and the particle radius of the *i*-th particle is *r*_i_ (1 ≤ *i* ≤ *n*), the equivalent particle diameter *D*_m_ of the particle clusters can be calculated by Eq. (9)^55^ as follows:

9 $$D_{m} = 2\sqrt {\sum\limits_{i}^{n} {r_{i}^{2} } }$$

The steps to gather particle size distribution are as follows:

1. The equivalent particle diameter* D*_m_ calculation: The equivalent particle diameter *D*_m_ is calculated by Eq. (9)
2. The size range calculation: The size range is determined by the maximum and minimum value of *D*_m_. The number of particle size group is set to 20 in this paper.
3. The percentage less than a particle size calculation: The percentage in quality is accumulated if the particle size is less than size of particle group.

**Figure 14:**
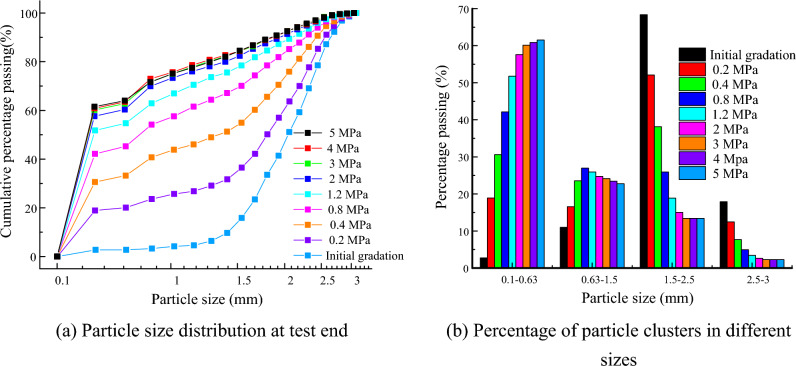
Evolution of particle size distribution.

Figure 14a presents the particle size distribution at the test end under different confining pressures., Even under the low confining pressure, the particle clusters are still broken. The number of broken particle clusters increases with an increase in the confining pressure, but the growth rate gradually decreases. Figure 14b presents the percentage of particle clusters of different sizes. The degree of crushing varies for different particle sizes. The percentage of particle clusters increases significantly in the size range of 0.1–0.63 mm. The maximum particle diameter of the particles in the clusters is 0.6 mm. This indicates that the crushing of the particle clusters easily breaks into a large number of single fine particles. The percentage of particle clusters first increases and then decreases with an increase in the confining pressure in the size range of 0.63–1.5 mm. The result indicates that when the confining pressure is large, small-size particle clusters will be broken. The percentage of particle clusters decreases significantly in the size range of 1.5–2.5 mm. This result is attributed to the fact that many particle clusters have crushing in this size group. The percentage of particle clusters decreases slightly in the size range of 2.5–3 mm. A more interesting finding is that there are still some large particle clusters that are not broken, even under high confining pressures. This phenomenon is explained by the hydrostatic effect^56^. The larger particles are protected by smaller particles around them, which makes them more resistive to particle crushing.

### Microcrack characteristics

**Figure 15:**
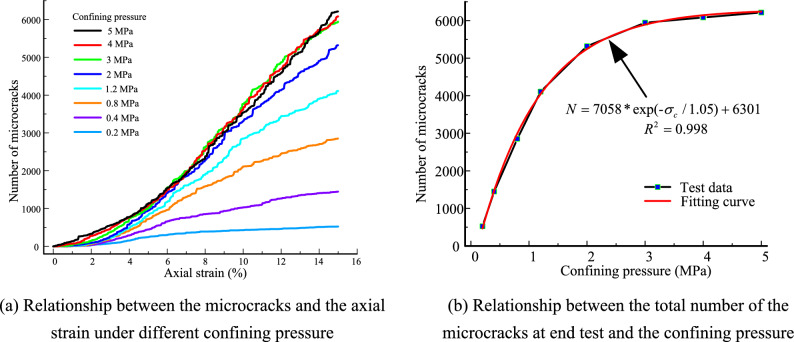
Evolution of the number of microcracks.

In the numerical model, a bond contact fracture produces a release of strain energy, i.e., a microcrack occurs. Figure 15a shows the relationship between the microcracks and the axial strain. For samples under confining pressure of 0.2 MPa and 0.4 MPa, the microcracks first increase and then remain constant or slightly increase for the samples. For samples under confining pressure of greater than 0.4 MPa, the microcracks keep increasing. When the confining pressure is less than 2 MPa, the growth rate of the microcracks first increases and then decreases with increasing axial strain. The distribution of the microcrack curves is scattered. When the confining pressure is greater than 2 MPa, the rate of increase of the microcracks first increases until axial strain reaches 3%, and then remain constant with an increasing axial strain. The distribution of the microcrack curves is observably concentrated. Figure 15b presents the relationship between the total number of microcracks at the test end and the confining pressure. The number of microcracks increases exponentially and the growth rate gradually decreases with an increase in the confining pressure. When the confining pressure reaches 3 MPa, the number of microcracks remains constant. This result indicates that there is functionally no correlation of particle crushing and stress level when the confining pressure is extreme due to the hydrostatic effect. This hydrostatic effect is growing since the number of small grains increases during the compress process. The hydrostatic environment will be established when a number of small grains crowd around larger particles. Therefore, the grains are difficult to break.

**Figure 16:**
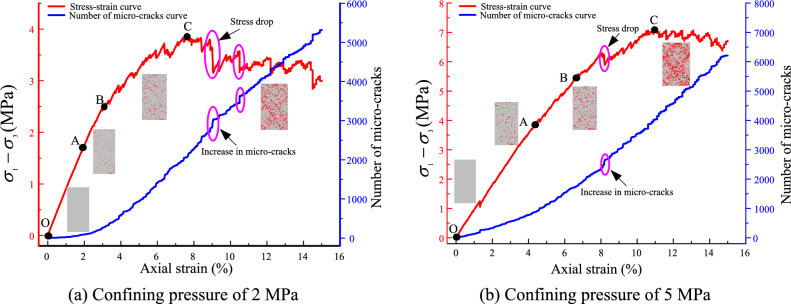
Relationship between the microcracks and the deviator stress.

To research the relationship between the microcracks and the deviator stress. The test results of samples under the confining pressure of 2 MPa and 5 MPa were selected, as shown in Fig. 16. The evolution of microcracks is affected by the loading deviator stress. The stress is an initial hardening and then a softening with axial strain. The evolution of the stress–strain curve was divided into four stages. In the OA stage, the stiff response is exhibited in this stage. The stress increases linearly with the axial strain. The mechanical behavior of the sample exhibited pure elasticity. No micro-cracks or a few micro-cracks occurred in the samples. In the AB stage, the microcracks increase slowly with the axial strain, and the distribution of the microcracks is sporadic. The stress gradually increases to point B, where yield occurs. In the BC stage, the microcracks increase rapidly with the axial strain. The stress increases from yield point B to peak point C. After point C, the number of microcracks increases continuously. The stress decreases in a wave manner. The strain softening is exhibited in the samples. The microcracks gradually run through and form broken banding in the samples. The evolution of the microcracks in the specimen can be classified into four phases: (1) initiation; (2) development; (3) extension and (4) arrest. When the microcracks interpenetrated, broken banding occurred in the sample. Additionally, compared to the evolution of the stress and microcracks, we find that an increase in microcracks results in a stress drop. This result indicates that the contact fracture weakened the mechanical properties of the sample.

### Evolution of crushing energy

Particle crushing is essentially the process of converting particle deformation energy to surface energy. According to Griffith's theory of fracture mechanics, the internal micro-cracks begin to expand when the material enters the plastic deformation under large external load. The material finally breaks as the microcracks expand and connect, which causes new surfaces to form. The load work is converted into nascent surface energy during the process. Therefore, the impact of particle crushing on engineering properties is the energy conversion^57^.

For the granular materials, there is an exchange and dissipation of energy during shearing^58,59^, including (1) particle crushing energy, (2) elastic deformation energy, (3) friction energy, (4) kinetic energy and (5) dissipation energy. Assuming that the total input energy was generated by the external force is *U*, Eq. (10) was obtained according to the first law of thermodynamics:

10 $$U = U_{{\text{e}}} + U_{{\text{k}}} + U_{{\text{f}}} + U_{{\text{d}}} + U_{{\text{b}}}$$

where $U_{{\text{e}}}$ is the elastic deformation energy; $U_{{\text{f}}}$ is the friction energy;$U_{{\text{k}}}$ is the kinetic energy; $U_{{\text{d}}}$ is the dissipation energy; and $U_{{\text{b}}}$ is the particle crushing energy.

In the 2-D numerical simulation test, the external force is provided by the upper and lower loading plates, and the total input energy *U* generated by the external force was obtained by Eq. (11) as follows:

11 $$U = \sum {(F_{1} \Delta {\text{U}}_{1} { + }F_{2} \Delta {\text{U}}_{2} } )$$

where $F_{1}$ is the external force of the upper plate; $F_{2}$ is the external force of the lower plate; $\Delta {\text{U}}_{1}$ is the displacement of the upper plate; and $\Delta {\text{U}}_{2}$ is the displacement of the lower plate.

The elastic deformation energy can be calculated by Eq. (12):

12 $$U_{{\text{e}}} = \frac{1}{2}\sum\limits_{{N_{c}^{l} }} {\left( {\frac{{(F_{i}^{n} )^{2} }}{{k_{n} }} + \frac{{(F_{i}^{s} )^{2} }}{{k_{s} }}} \right)} + \frac{1}{2}\sum\limits_{{N_{{_{c} }}^{b} }} {\left( {\frac{{(\overline{F}_{i}^{n} )^{2} }}{{\overline{k}_{n} \overline{A}}} + \frac{{(\overline{F}_{i}^{s} )^{2} }}{{\overline{k}_{s} \overline{A}}} + \frac{{\overline{M}^{2} }}{{\overline{k}_{n} \overline{I}}}} \right)}$$

where $N_{c}^{l}$ is the number of linear contacts; $F_{i}^{n}$ is the normal force of linear contact; $F_{i}^{s}$ is the tangential force of linear contact; $k_{n}$ is the linear normal stiffness; $k_{s}$ is the linear tangential stiffness; $N_{c}^{b}$ is the number of parallel bond contacts; $\overline{F}_{i}^{n}$ is the normal force of parallel bond contact; $\overline{F}_{i}^{s}$ is the tangential force of parallel bond contact; $\overline{k}_{n}$ is the parallel bond normal stiffness; $\overline{k}_{s}$ is the parallel bond tangential stiffness; $\overline{A}$ is the cross-sectional area; $\overline{M}$ is the parallel bond moment; and $\overline{I}$ is the moment of inertia of parallel bonds.

The kinetic energy can be calculated by Eq. (13):

13 $$U_{{\text{k}}} = \frac{1}{2}\sum\limits_{{N_{p} }} {\left[ {m_{i} (U_{i}^{c} )^{2} + I_{i} (\theta_{i}^{c} )^{2} } \right]}$$

where $N_{p}$ is the number of particles; $m_{i}$ is the mass of the particle *i*; $I_{i}$ is the moment of inertia of the particle *i*; $U_{i}^{c}$ is the translational velocity of the particles; and $\theta_{i}^{c}$ is the rotational velocity of the particles.

The friction energy can be calculated by Eq. (14):

14 $$U_{{\text{f}}} = \sum\limits_{{N_{c} }} {(F_{i}^{s} } \Delta {\text{U}}_{i}^{s} )$$

where $F_{i}^{s}$ is the tangential force of linear contact; $\Delta {\text{U}}_{i}^{s}$ is the increment of displacement; and $N_{c}$ is the number of particle contacts.

The dissipation energy can be calculated by Eq. (15):

15 $$U_{{\text{d}}} = \sum\limits_{{N_{p} }} {(F_{i}^{d} \Delta {\text{U}}_{i}^{d} } + F_{i}^{dp} \Delta {\text{U}}_{i}^{dp} )$$

where $F_{i}^{d}$ is the local damping force of the particles; $\Delta {\text{U}}_{i}^{d}$ is the displacement increment of the particle; $F_{i}^{dp}$ is the viscous damping force of the particles; and $\Delta {\text{U}}_{i}^{dp}$ is the relative displacement increment of the particles.

**Figure 17:**
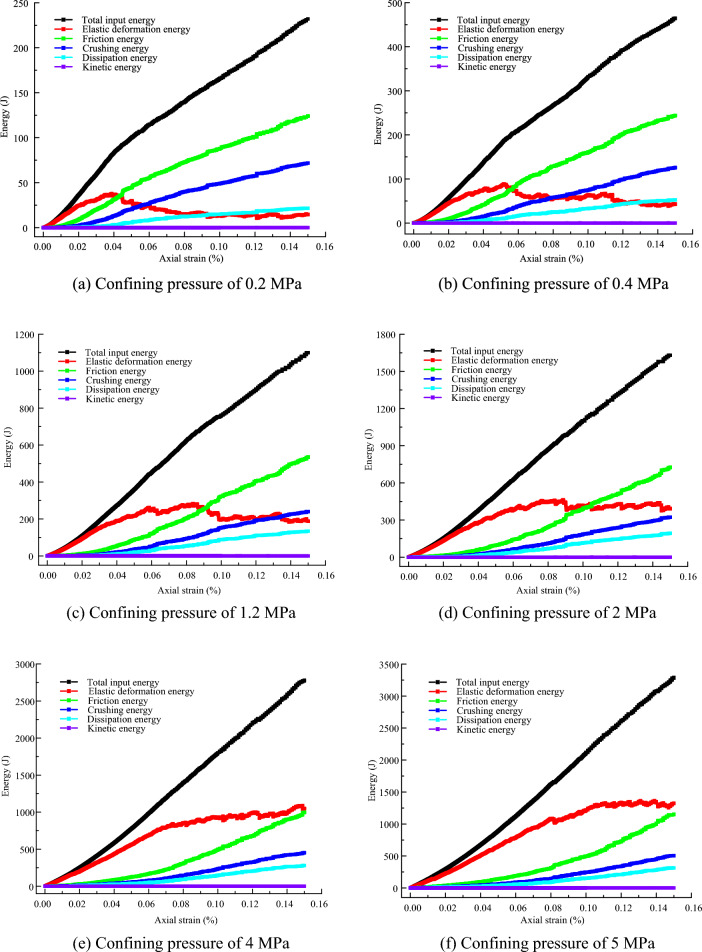
Evolution of the energies.

By substituting Eqs. (11)–(15) into Eq. (10), the particle crushing energy can be calculated. Figure 17 presents the evolution of the energies. When the axial strain is less than 3%, the total energy is stored in the form of elastic deformation energy. The mechanical behavior of the samples is elastic. When the axial strain is greater than 3%, the friction energy and particle crushing energy increase with axial strain due to particle movement and particle breakage. Due to the effects of dissipation energy, the kinetic energy remains zero. This result verified that the sample was quasi-static during the shear process, and the calculation results were credible. Furthermore, the total energy, frictional energy, crushing energy, and dissipation energy all increase with the axial strain. The elastic deformation energy first increases and then decreases or keeps constant with the axial strain. The reasons are that the stored elastic deformation energy will be released and transformed into other forms of energy due to the effects of strain softening at a large axial strain.

**Figure 18:**
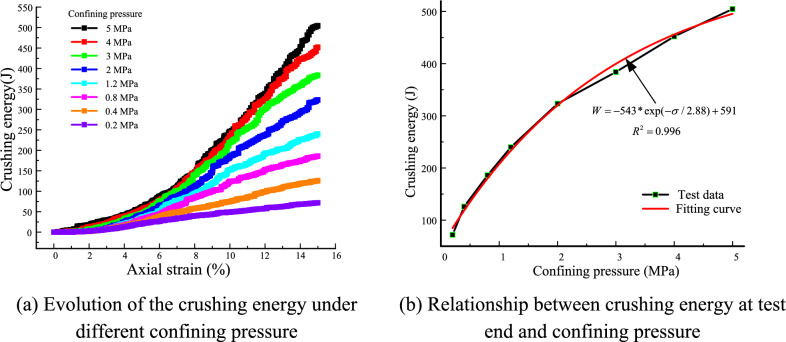
Evolution of the crushing energy.

Figure 18a presents the evolution of the crushing energy under different confining pressures. The crushing energy increases with the axial strain. Furthermore, the growth speed of crushing energy increases with increasing confining pressure. Compared with Fig. 15a, we can see that the evolution of the crushing energy curves is consistent with the change in the number of microcracks under different confining pressures. This result is attributed to the crushing energy coming from the fracture of the contacts.

Many quantitative research methods have been proposed to study the particle breakage. The most widely used is the breakage index *B*_r_ proposed by Bardin according to the change of particle size distribution. However, the theoretical basis of breakage index *B*_r_ is insufficient because of energy dissipation in crushing is not considered. The breakage degree of sand was studied using the energy method by Ueng et al.^60^. The particle crushing is the formation of penetrate cracks, which is the process of the energy consumption. Therefore, the crushing energy is used to respresent the particle breakage in this paper. Figure 18b presents the relationship between the crushing energy at the test end and the confining pressure. The crushing energy at the end of the test increases exponentially with increasing confining pressure, and growth rate gradually decreases. This result indicates that although irregular granular materials have a large degree of crushing, the potential crushing ability is low under high confining pressures. This result is consistent with the concept of crushing potential proposed by Einav^61^, i.e., when the confining pressure reaches a certain extreme value, irregular granular materials will reach a stable gradation and further crushing will be tiny or not emerge^62^.

## Conclusion

Based on the polygon construction principle, particle clusters with irregular sides were modelled using a DEM with a large number of particles. A biaxial numerical model test was carried out under the low and high confining pressures. The influence of the stress level on the mechanical behavior and particle crushing of granular materials was studied in this paper. The main conclusions were given as follows:

1. For samples under the low confining pressures, there is a significant stress drop with dilatancy. For samples under high confining pressures, there is only a slight or no stress drop with contraction. The shear strength shows a significantly nonlinear behavior and increases with the confining pressure due to particle crushing.
2. Macroscopic shear banding at a dip angle of 45° occurs in samples under low confining pressures. The fracture zone in the samples is in the form of an ‘X’ when the confining pressure is high. As the confining pressure increases, particle movement is transformed from particle rotation into particle breakage, and the sample deformation is transformed from macroscopic shear banding to macroscopic broken banding. Comparing the distribution of force chains, strong force chains in samples under low confining pressures are sparser than those under high confining pressures.
3. The crushing of particle clusters is attributed to the contact fracture of internal adhesion. The fragmentation of particle clusters increases with increasing axial strain. The number of contact fractures increases with an increase in the confining pressure. The particle clusters easily break into many single fine particles when the confining pressure is high, which leads to the refinement of the particle size distribution.
4. The first law of thermodynamics was used to analyse the evolution of the crushing energy, which represents the breakage degree of particle in the shear process. The crushing energy increases with the axial strain. The crushing energy at the end of the test increases exponentially with increasing confining pressure. When the confining pressure reaches a certain extreme value, granular materials will reach a stable gradation and further crushing will be tiny or not emerge.

## Data Availability

The datasets used and/or analysed during the current study available from the corresponding author on request.
